# The role of mitochondria in the pathogenesis of Kawasaki disease

**DOI:** 10.3389/fimmu.2022.1017401

**Published:** 2022-10-10

**Authors:** Mikayla A. Beckley, Sadeep Shrestha, Keshav K. Singh, Michael A. Portman

**Affiliations:** ^1^ Center for Integrative Brain Research, Seattle Children’s Research Institute, Seattle, WA, United States; ^2^ Department of Epidemiology, School of Public Health University of Alabama at Birmingham, Birmingham, AL, United States; ^3^ Department of Genetics, Heersink School of Medicine, University of Alabama at Birmingham, Birmingham, AL, United States; ^4^ Department of Pediatrics, Division of Cardiology, University of Washington, Seattle, WA, United States

**Keywords:** mitochondria, mitochondrial DNA, inflammation, inflammasome, Kawasaki disease, reactive oxygen species, mitophagy

## Abstract

Kawasaki disease is a systemic vasculitis, especially of the coronary arteries, affecting children. Despite extensive research, much is still unknown about the principal driver behind the amplified inflammatory response. We propose mitochondria may play a critical role. Mitochondria serve as a central hub, influencing energy generation, cell proliferation, and bioenergetics. Regulation of these biological processes, however, comes at a price. Release of mitochondrial DNA into the cytoplasm acts as damage-associated molecular patterns, initiating the development of inflammation. As a source of reactive oxygen species, they facilitate activation of the NLRP3 inflammasome. Kawasaki disease involves many of these inflammatory pathways. Progressive mitochondrial dysfunction alters the activity of immune cells and may play a role in the pathogenesis of Kawasaki disease. Because they contain their own genome, mitochondria are susceptible to mutation which can propagate their dysfunction and immunostimulatory potential. Population-specific variants in mitochondrial DNA have also been linked to racial disparities in disease risk and treatment response. Our objective is to critically examine the current literature of mitochondria’s role in coordinating proinflammatory signaling pathways, focusing on potential mitochondrial dysfunction in Kawasaki disease. No association between impaired mitochondrial function and Kawasaki disease exists, but we suggest a relationship between the two. We hypothesize a framework of mitochondrial determinants that may contribute to ethnic/racial disparities in the progression of Kawasaki disease.

## 1 Introduction

Kawasaki disease (KD) is a pediatric systemic vasculitis of unknown etiology. The systemic inflammatory response is self-limited, but local coronary artery inflammation can continue and result in persistent coronary artery wall abnormalities. The mechanisms still require clarification. Coronary artery aneurysms (CAA) may develop in children with KD even with treatment, leading to long-term cardiovascular complications into adulthood (1). Diagnosis of KD is based on clinical features established by the American Heart Association (2). In addition to prolonged fevers (≥5 days), KD is characterized by conjunctival injection, rash, swelling of the hands and feet, cervical lymphadenopathy, and oral mucosal changes (strawberry tongue and red, cracked lips), but clinical presentation is quite variable (2). Incidence of KD varies across the globe, with Japan reporting an annual incidence in 2016 of approximately 309 cases of KD per 100, 000 children under the age of five (3) and about 19.8 per 100, 000 children of the same age group in the United States during 2016 (https://www.cdc.gov/kawasaki/about.html).

KD is complex with both potential genetic and environmental factors contributing to disease progression. The trigger initiating the hyper-inflammatory response requires clarification. Published studies demonstrate that mitochondria are critical drivers of inflammation by initiating activation of inflammasomes and regulating immune cell functions (4–6). Alterations in mitochondrial activity and mitophagy can result in imbalances in cell bioenergetics and secretory dysfunction, which can cause dysregulation of inflammatory processes.

## 2 Mechanisms inducing inflammation

### 2.1 NLRP3 inflammasome

Molecular mechanisms contributing to the inflammatory response in KD remains unknown, but emerging evidence suggests that inflammasomes may play a key role during the acute phase. Inflammasomes, intracellular, multi-protein signaling complexes of the innate immune system, are critical activators and controllers of the inflammatory response. Of special interest is the *NOD-, LRR-, and pyrin domain-containing 3* (*NLRP3*) inflammasome as it is associated with antimicrobial responses and several inflammatory disorders, including KD (7–11).

NLRP3 is a cytosolic protein stimulated in response to pathogen-associated molecular patterns (PAMPs) and host-derived danger associated molecular patterns (DAMPs). Pattern recognition receptors (PRRs) expressed primarily in monocytes/macrophages recognize these danger signals (11). Activation of the NLRP3 inflammasome recruits caspase-1, which then cleaves pro-IL-1β and pro-IL-18 into their active forms, IL-1β and IL-18, respectively (11). These proinflammatory cytokines subsequently initiate and amplify pathways that promote inflammation (11).

Dysregulated activation of the NLRP3 inflammasome may play a critical role in KD. The *Candida albicans* water-soluble fraction (CAWS) murine model of KD activates NLRP3 and expression of IL-1β (7). Inflammation mediated by IL-1β is also observed in the *Lactobacillus casei* cell wall extract (LCWE) murine model of KD vasculitis (8, 9). Further, increased expression levels of NLRP3, activated caspase 1, and IL-1β are present in sera from patients with KD compared to healthy controls (10). Using the CAWS murine model, addition of an inhibitor that specifically targets NLRP3 and prevents its oligomerization significantly decreased NLRP3, caspase 1, and IL-1β protein expression levels (10). These mice and human studies support a relationship between the activation of NLRP3 inflammasomes and immune dysfunction in KD, but they do not clearly define what triggers the activation of NLRP3 in these models.

Mitochondrial dysfunction activates NLRP3 inflammasomes (4–6). Activating stimuli leading to formation of the NLRP3 inflammasome includes its relocalization to the mitochondria and the cytosolic presence of mitochondrial derived ROS (mROS) and mitochondrial DNA (mtDNA) (5, 11). Malfunctioning mitochondria produce amplified levels of mROS, which causes further damage to the organelle (6, 12) due to the proximity of mtDNA to oxidative phosphorylation machinery and lack of introns, histone protection, and DNA repair mechanisms (13, 14). Oxidative stress increases calcium concentration within the inner mitochondrial membrane, which opens the mitochondrial permeability transition pore and releases mtDNA into the cytosol where it promotes activation of the NLRP3 inflammasome (6, 15). Thus, increased presence of mitochondrial DAMPs aberrantly favor activation of NLRP3 inflammasomes and may drive the dysregulated inflammatory response in KD.

### 2.2 Mitophagy

Another fundamental driver of NLRP3-induced inflammation is the failure of autophagy and mitophagy processes (16). Mitophagy, an autophagy-specific degradation process, removes dysfunctional mitochondria (17). During the acute phase of KD, decreased mRNA levels of autophagy markers are present and are significantly increased after treatment with IVIG compared to controls (18). In the LCWE murine model of KD vasculitis, Marek-lannucci et al. observed reduced clearance of damaged mitochondria. This was reflected by decreased autophagic flux as well as increased expression of mTOR pathway-related proteins, leading to inhibition of autophagy (19).

Effective clearance of dysfunctional mitochondria is critical for cell survival, especially in tissues with high energy demands (i.e., cardiovascular tissues). When mitophagy is impaired, dysfunctional mitochondria accumulate and produce greater amounts of mROS compared to healthy organelles (20), which stimulates activation of the NLRP3 inflammasome. Thus, defective mitophagy may propagate the hyperinflammatory response in KD through the accumulation of malfunctioning mitochondria.

### 2.3 Viral and bacterial infection

While the etiology of KD remains uncertain, data suggest KD is an immunologic response triggered upon exposure to an infectious agent, such as a virus or bacteria, or environmental antigen (21). For regions across the Northern Hemisphere, occurrences of KD peak in the winter with low cases reported during the summer (22). This type of seasonal variation is also a characteristic of several infectious diseases, including influenza and meningitis (23).

In addition to this type of seasonal variation, infections have also been directly related to an elevated risk in KD onset or presentation with similar symptoms. Prior hospitalization for a bacterial illness in a 2012 Washington state study was associated with a 2.8-fold increased risk of developing KD (24). Superantigens from bacteria or viruses may also contribute to the onset of disease (25). Prior bacterial (with or without antibiotic treatment) or viral infection might alter mitochondrial dynamics in immune cells leading to greater susceptibility to KD.

#### 2.3.1 Viruses

**Figure 1:**
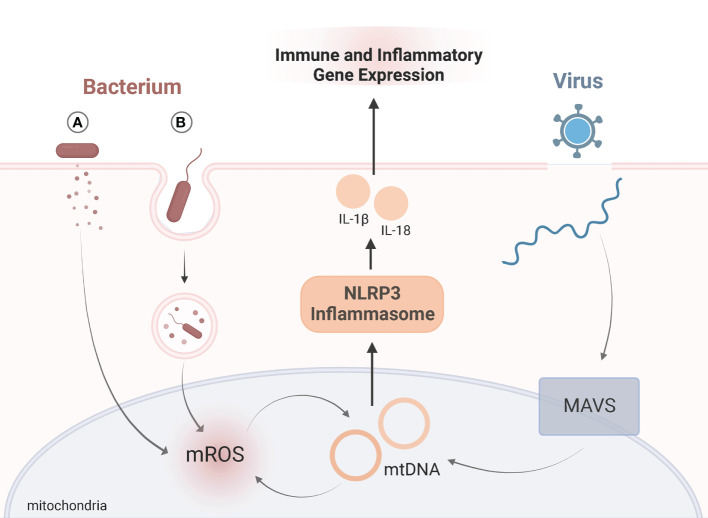
Viral and Bacterial Targeting of MitochondriaSchematic representation of role of mitochondria in immune signaling upon viral (blue) or bacterial (red) infection. Exposure to viruses or bacteria both activate immune responses by altering mitochondrial dynamics, namely the NLRP3 inflammasome (orange). Upon viral infection, mitochondrial antiviral-signaling protein (MAVS) is located in the outer mitochondrial membrane and serves as an important signaling. MAVS triggers release of mtDNA which activates the NLRP3 inflammasome. Bacteria manipulate mitochondria during infection by either **(A)** secreting bacterial proteins or toxins, or **(B)** entering host cells *via* phagocytosis where mitochondria are recruited to the phagosome through toll-like receptor (TLR) signaling. Bacteria can either suppress or amplify mROS generation. The antimicrobial activity of mROS influences mtDNA “leakage”, which further amplifies mROS, and activation of the NLRP3 inflammasome. For both viral and bacterial infections, activation of the NLRP3 inflammasome triggers the release of IL-1β and IL-18. Release of these proinflammatory cytokines influences immune and inflammatory gene expression. Adapted from “Endocytosis and Exocytosis with Membrane Rupture (Layout)”, by BioRender.com. Retrieved from https://app.biorender.com/biorender-templates.

Viruses evade mitochondria-mediated immune responses by directly or indirectly interacting with host mitochondria. Hijacking mitochondrial functions allows viruses to persist and proliferate (26). The mitochondrial antiviral-signaling protein (MAVS) is located in the outer mitochondrial membrane and serves as an important signaling platform in viral infections (27, 28). Upon infection MAVS promotes NLRP3 inflammasome activation, which influences immune responses (29) (**Figure 1**). While the role of mitochondrial dynamics in viral infections is still evolving, several viruses impair mitophagy and induce mtDNA release, such as hepatitis B and C virus, measles, and coxsackievirus B (30–33). Influenza virus downregulates innate immune responses by suppressing MAVS and the NLRP3 inflammasome pathway (34). Infection with SARS-CoV-1 also suppresses immune responses by targeting and degrading MAVS (35, 36). Thus, viral manipulation of mitochondria may contribute to the hyper-inflammatory response in KD.

#### 2.3.2 Bacteria

Bacteria have developed multiple strategies to subvert mitochondrial-mediated antimicrobial responses. Like viruses, they can manipulate mitochondria indirectly or directly by secreting toxins or entering host cells, respectively (37). Extracellular bacteria can induce mitochondrial damage by injecting bacterial proteins (as is the case with *enteropathogenic Escherichia coli*) *(* 38) or by secreting pore-forming toxins (like with *Staphylococcus aureus*) (37). Through phagocytosis, a defense mechanism designed to engulf pathogens, bacteria can enter host cells (39). Bacteria serve as a source of PAMPs and are recognized by transmembrane toll-like receptors (TLRs). Recognition of PAMPs through TLR signaling triggers the recruitment of mitochondria to the phagosome. To clear an infection, mROS are generated (39) (**Figure 1**). In human neutrophils, mitochondria play a critical role in killing *Staphylococcus aureus (* 40 *).* Jiao et al., reported damaged mitochondria and abnormal accumulation of mROS in response to a *Yersinia pestis (Y. pestis*) infection (41). Without functional mitophagy processes, mitochondria infected with *Y. pestis* persisted (41). Thus, bacterial infection may manipulate mitochondrial function and promote inflammatory responses contributing to the onset of KD.

Additionally, KD patients are frequently exposed to antibiotics in the course of their illness. Multiple studies show high prevalence of antibiotic use in KD patients due to preceding bacterial illness or misdiagnosis (42–44). Some studies suggest that alterations in gut mucosa are responsible for onset of KD in patients exposed to antibiotics (44). However, emerging data show specifically that bactericidal beta-lactams, which are the most frequent antibiotics prescribed in children prior to KD (42), cause mitochondria dysfunction (45). Bactericidal antibiotic-induced effects lead to oxidative damage to mitochondria, DNA, proteins, and membrane lipids (45). Considering the potential involvement of dysfunctional mitochondria in KD pathogenesis, use of antibiotics may further compromise already impaired mitochondria.

### 2.4 Immune and non-immune cells

Dysregulated immune cell responses drive the acute phase of KD (2). Immune and non-immune cells, including macrophage/monocytes, neutrophils, and endothelial cells, express PRRs and are activated in response to PAMPs and DAMPs (46, 47). Endothelial cells are critical for the maintenance of vascular homeostatsis (46) and play a critical role in KD vasculitis and formation of coronary artery aneurysms (2, 48, 49). Upon activation, they recruit circulating inflammatory cells and increase vascular cell wall permeability (50).

Mitochondria may also play a critical role in this inflammatory process, and when dysfunctional, may exacerbate these responses. A study using mice showed that mROS generation mediates activation of inflammasomes within immune cells and triggers vascular inflammation (51). Specifically, mROS within adventitial macrophages activated the NLRP3 inflammasome and initiated abdominal aortic aneurysm formation (51). Thus, impaired mitochondria may generate heightened production of mROS and activation of the NLRP3 inflammasome, leading to risk of coronary aneurysm formation in KD vasculitis.

**Figure 2:**
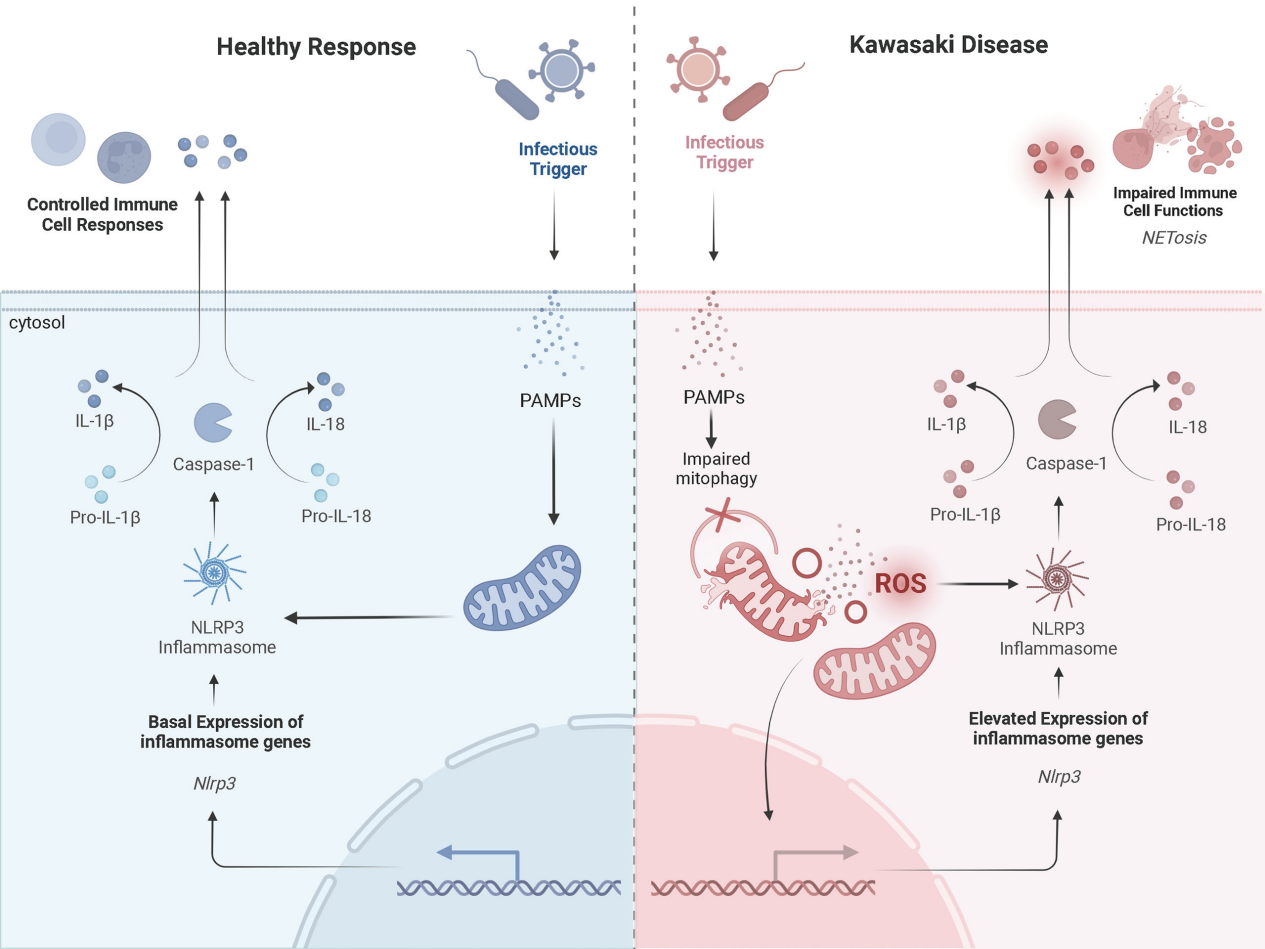
A Prospective Comparison of a Healthy Inflammatory Response and that in Kawasaki DiseaseProspective mechanisms of mitochondrial dysfunction and exaggerated activation of NLRP3 inflammasomes in KD upon infection compared to a healthy response. The healthy response is in blue, on the left-hand side of the figure. The KD response is in red, on the right-hand side of the diagram. Formation of the NLRP3 inflammasome complex occurs in the cytosol of monocytes/macrophages in the presence of PAMPs and/or DAMPs (i.e., cytosolic mROS and mtDNA). A healthy inflammatory response upon infection involves appropriate signaling from the mitochondria and activation of the NLRP3 inflammasome to clear the infection. In KD, damaged mitochondria release more mROS and mtDNA into the cytosol, which exaggerates activation of the NLRP3 inflammasome. This promotes NETosis and impairs immune cell functions. *Adapted from “Suppression of Inflammasome by IRF4 and IRF8 is Critical for T Cell Priming”, by www.BioRender.com (2022). Retrieved from https://app.biorender.com/biorender-templates*.

Further, neutrophils of patients with KD are more likely to form neutrophil extracellular traps (NETs) compared to healthy controls (52). NETs are web-like structures containing oxidized DNA, which stimulates pro-inflammatory responses and signaling pathways (52). For patients with KD, NETs suppressed apoptosis and exaggerated cytokine production (52). The factor initiating NET formation in KD is not entirely clear. Mitochondria are known inducers of NET formation and may explain why increased NETs are associated with KD vasculitis. For individuals with systemic lupus erythematosus (SLE), mROS trigger aberrant NET formation (53). In biopsy samples of lupus nephritis, endothelial damage was associated with increased release of mtDNA and NET formation (54). Similar findings for patients with granulomatous disease have also been observed (55). These findings highlight the role of mitochondria in the generation of NETs and release of stimuli known to activate the NLRP3 inflammasome. Thus, exaggerated signals derived from damaged mitochondria may increase the accumulation of NETs and drive the hyperinflammatory response in KD (**Figure 2**).

### 2.5 Mito-nuclear crosstalk

**Figure 3:**
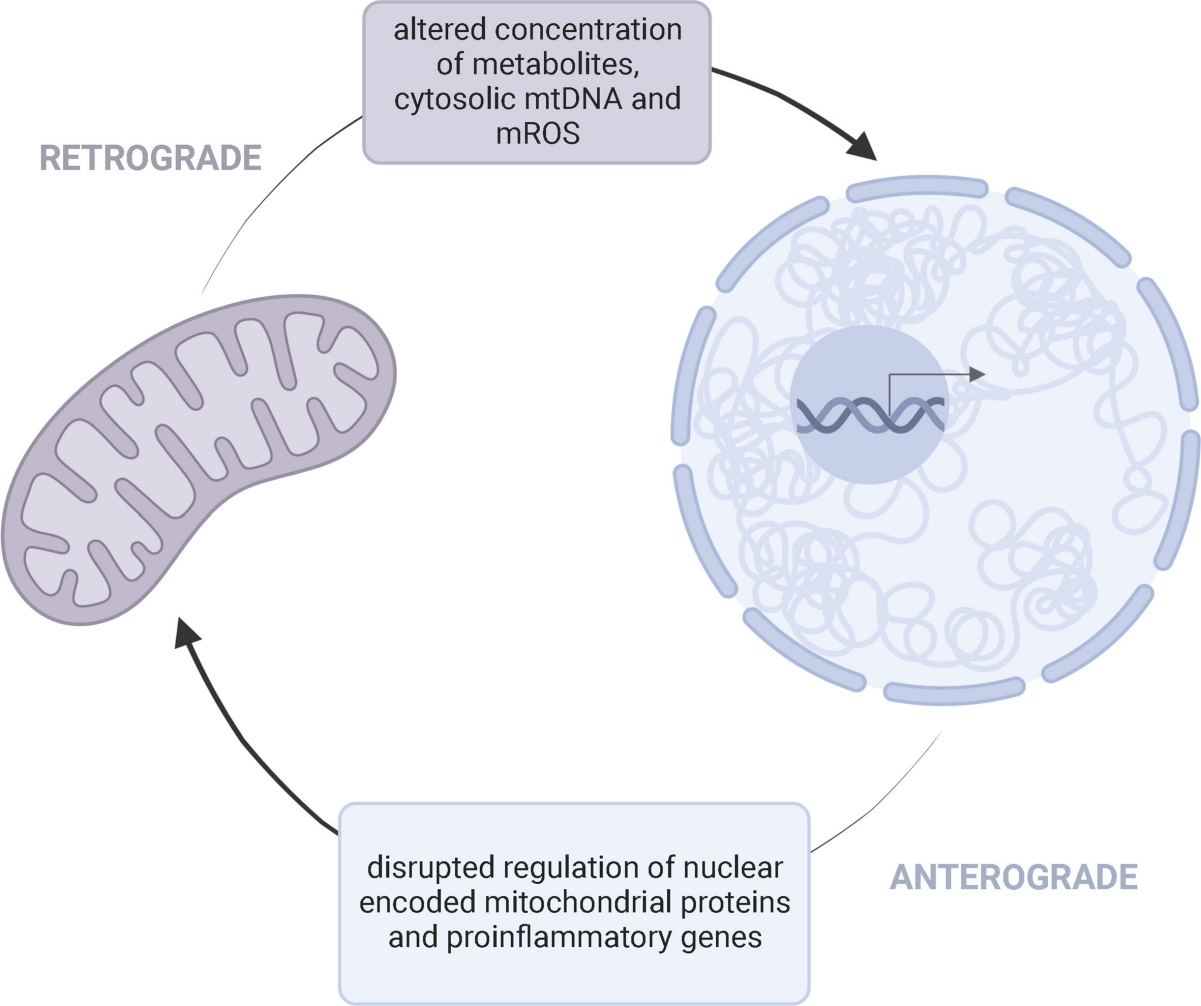
Bidirectional Mito-Nuclear CrosstalkMitochondria and the nucleus communicate through retrograde (purple) and anterograde (blue) signaling pathways. This bidirectional crosstalk allows a cell to maintain homeostasis and adapt to various pressures. Retrograde (mitochondria-to-nucleus) communication influences the regulation of nuclear encoded mitochondrial proteins and expression of proinflammatory genes through altered concentration of metabolites, cytosolic mtDNA, and mROS. Anterograde signaling, as a response, can further influence the availability of mitochondrial derived metabolities needed for cell survival and disrupt mitochondrial oxidative efficiency.

Through metabolite generation, mitochondria control the establishment, function, and maintenance of immune cells (56, 57). Mitochondria contain their own genome, but the nucleus encodes a majority of the proteins required for mitochondrial function (12). Thus, compromised mitochondria may result from defects in either the nuclear or mitochondrial genome. Children with mitochondrial disease primarily harbor pathogenic variants in nuclear DNA (nDNA) (58). “Crosstalk” between the mitochondrial and nuclear genome may potentially link the dynamic interplay between potential genetic and environmental factors contributing to KD onset/progression (**Figure 3**).

DNA methyltransferases (DNMTs) establish methylation patterns which reduces expression of proteins by inhibiting transcription factors from binding (59). Compared to controls, several genes are hypomethylated during the acute phase of KD (60). Levels of DNMT1 and DNMT3A are also significantly lower in patients with KD (61). Thus, suppressed epigenetic markers in patients with KD may increase expression of immune related genes. Why patients with KD experience epigenetic down-regulation of immune related genes is unclear, but mitochondrial metabolites and secreted factors may influence these transcriptional changes.

Variants in mtDNA may alter metabolic pathways critical for appropriate methylation patterns, while disrupting the concentration of mROS and cytosolic mtDNA. Mitochondria regulate nuclear epigenetics through metabolites, like S-adenosyl methionine (SAM). Histone methyltransferases use SAM as a precursor in methyl group transfer (12). In cell lines containing identical nuclei, but varying mtDNA contents, differences in methylation markers and expression of nuclear genes involved in inflammation is observed (56). Oxidative stress also acts as a signaling mediator, directly altering histone methylation profiles (62). At the same time, changes in nuclear epigenetics alter mitochondrial function. DNA hypomethylation increases the expression of nuclear-encoded mitochondrial genes, such as DNA polymerase gamma (*POLG*), which subsequently increases mtDNA content (63). This suggests a bidirectional crosstalk between both genomes is critical for maintaining appropriate gene expression and metabolic responses. A disruption in this mito-nuclear crosstalk may promote exaggerated expression of immune cells through impaired metabolite secretion and excessive oxidative damage.

## 3 Racial and ethnic disparities

The substantial racial and ethnic variation in KD incidence is another interesting feature. Epidemiological studies show a distinct disparity between Asian, African American, and other ethnicities in the prevalence of KD (2). Eastern Asian countries continuously report a substantially higher incidence of KD than Western countries. Even the incidence in American children of Japanese or African American descent significantly exceeds that of Caucasian descent (64). Compared with Caucasian children, African American children with KD have more severe inflammation and extended hospitalizations (65). Previous reports attributed these racial disparities to differences in socioeconomic and environmental factors (66). However, these disparities may also be due to genetic differences (67), including variations in the mitochondrial genome. Alterations in the mitochondrial genome contribute to variations in function and metabolic capacities. Owing to the proximity to the ETC, mtDNA is constantly exposed to the effects of ROS creating susceptibility to mutational formations. The absence of histone protection and lack of DNA damage repair mechanisms (13, 14) also results in a higher accumulation of mtDNA mutations (about 100-1000 fold) than those in the nuclear genome (12). These mutations tend to have more consequences than mutations in nuclear DNA (nDNA) (68). As humans migrated across the globe, ancient variants accumulated as an adaptation tool to regional selective pressures (12). These conserved mtDNA mutations generally provided superior adaptation to the environment and overtime defined functionally distinct mitochondrial haplogroups (12) The African mtDNA haplogroup (L) serves as the most ancient, giving rise to other haplogroups as populations migrated to other continents (69). Once adaptive, these haplogroups now serve as biomarkers for racial susceptibility to disease (12). Thus, mitochondria harboring certain haplotypes may amplify ROS production and inflammation (70–72).

Kenney and colleagues characterize this relationship by illustrating how cells harboring the L haplogroup (African origin) differentially mediate the expression of genes in inflammatory pathways (71). In a Taiwanese cohort, those with higher SLE disease activity and nephritis belonged to the D130 haplogroup (73). These findings, along with others, demonstrate the importance of mtDNA haplogroups in mediating disease severity (74–76).

In addition to haplogroup-associated studies, several researchers highlight disease risk with specific mtDNA single nucleotide polymorphisms (SNPs), further defining haplogroups into haplotypes (12). In a multi-ethnic cohort study, the missense 4917 SNP in the ND2 gene conferred risk of colorectal cancer in European-Americans, but not Africans, Asians, or Latinos (68). Thus, mitochondrial haplotypes may differentially mediate mROS production and the expression of nuclear genes involved in the inflammatory pathways critical in KD.

## 4 Prospective mitochondrial two-hit model in Kawasaki disease

The complex nature of KD suggests the involvement of several genetic and environmental factors. As described above, mutations in the mitochondrial genome contribute to several inflammatory disorders. In a vicious cycle, impaired mitochondria trigger an increase in mROS production. An increase in mROS activates the NLRP3 inflammasome and release of IL-1β. At the same time, these free radicals oxidize lipids and cause mutations that may further impair mitochondrial function, including reduced mitophagy responses (6, 12). Viral and bacterial infections also impair mitophagy, which further influences the abnormal functioning of immune cells.

**Figure 4:**
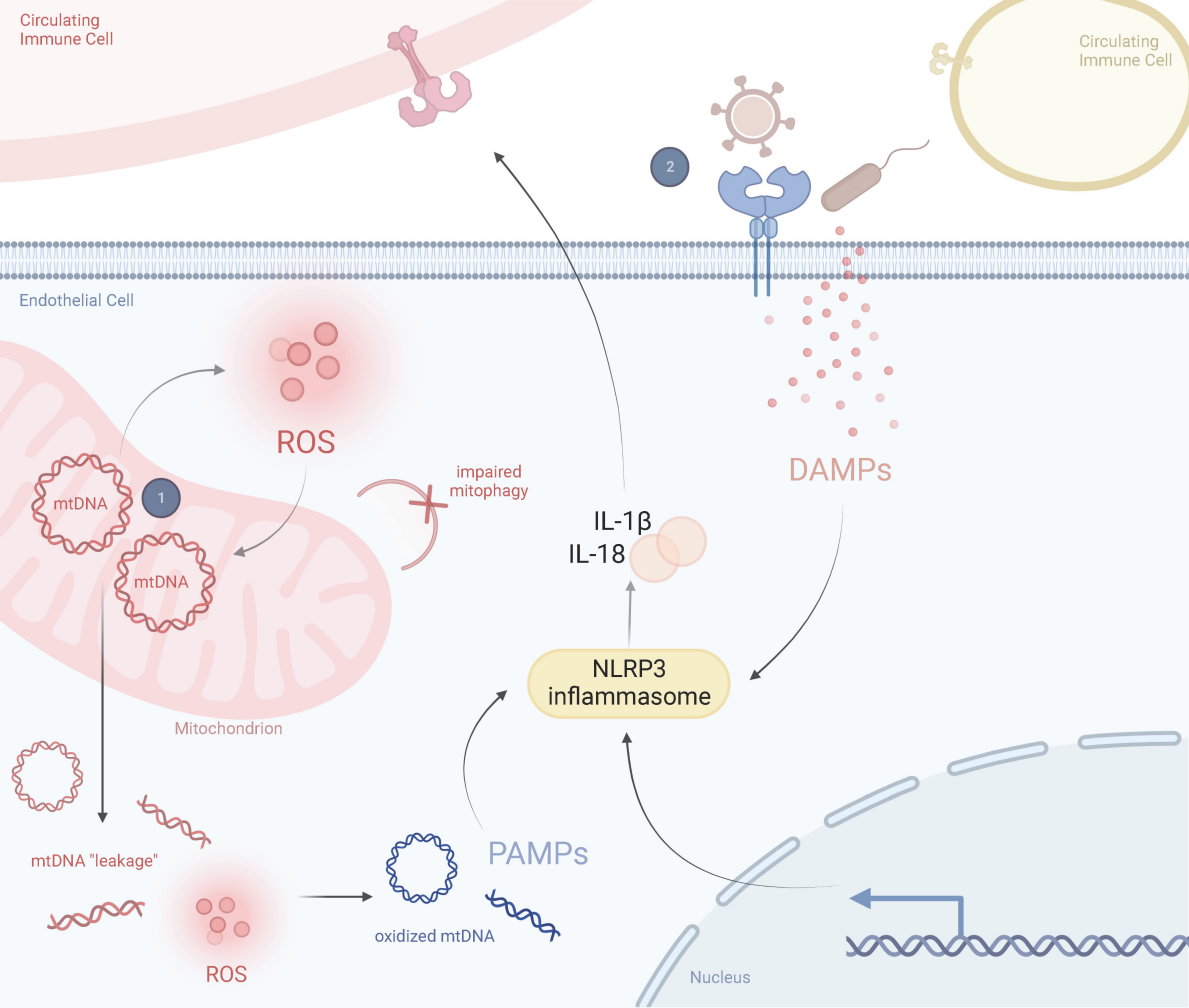
Prospective Two-Hit Model of Mitochondrial Dysfunction in Kawasaki DiseaseSchematic representation of the perspective inflammatory response in a healthy state and in a Kawasaki diseased state, including the role of mitochondria in driving KD responses. (1) A mitochondrial SNP or haplogroup background results in abnormally proinflammatory mitochondria that are more likely to release mtDNA into the cytosol and produce ROS. These ROS oxidize cytosolic mtDNA and are recognized by circulating immune cells as DAMPs. Exposure to DAMPs activates the NLRP3 inflammasome, leading to proinflammatory cytokine release, such as IL-1β and IL-18. (2) Several viruses or bacteria manipulate mitochondria during infection. This could lead to a hyperimmune response and cytokine storm from previously damaged mitochondria (i.e., those prone to pyroptosis or impaired mitophagy). ROS and secreted cytokines further amplify this response in a feedback loop. Adapted from “Lipids and Proteins Involved in Lipid Uptake and Metabolism in Cardiac Lipotoxicity”, by BioRender.com. Retrieved from https://app.biorender.com/biorender-templates.

Thus, we propose a two-hit model for KD pathogenesis where a mitochondrial polymorphism or haplogroup, or a mutation within the nuclear genome that impairs mitochondrial function, predisposes endothelial cell mitochondria to mtDNA “leakage”. These mutant mitochondria are susceptible to infectious agents and are further compromised during infection, resulting in a dysfunctional state including exaggerated activation of NLRP3 inflammasomes and release of proinflammatory cytokines. This then influences the expression of circulating immune cells, including macrophages/monocytes and neutrophils, which further amplifies the inflammatory response (**Figure 4**).

### 4.1 Background of hit one

**Figure 5:**
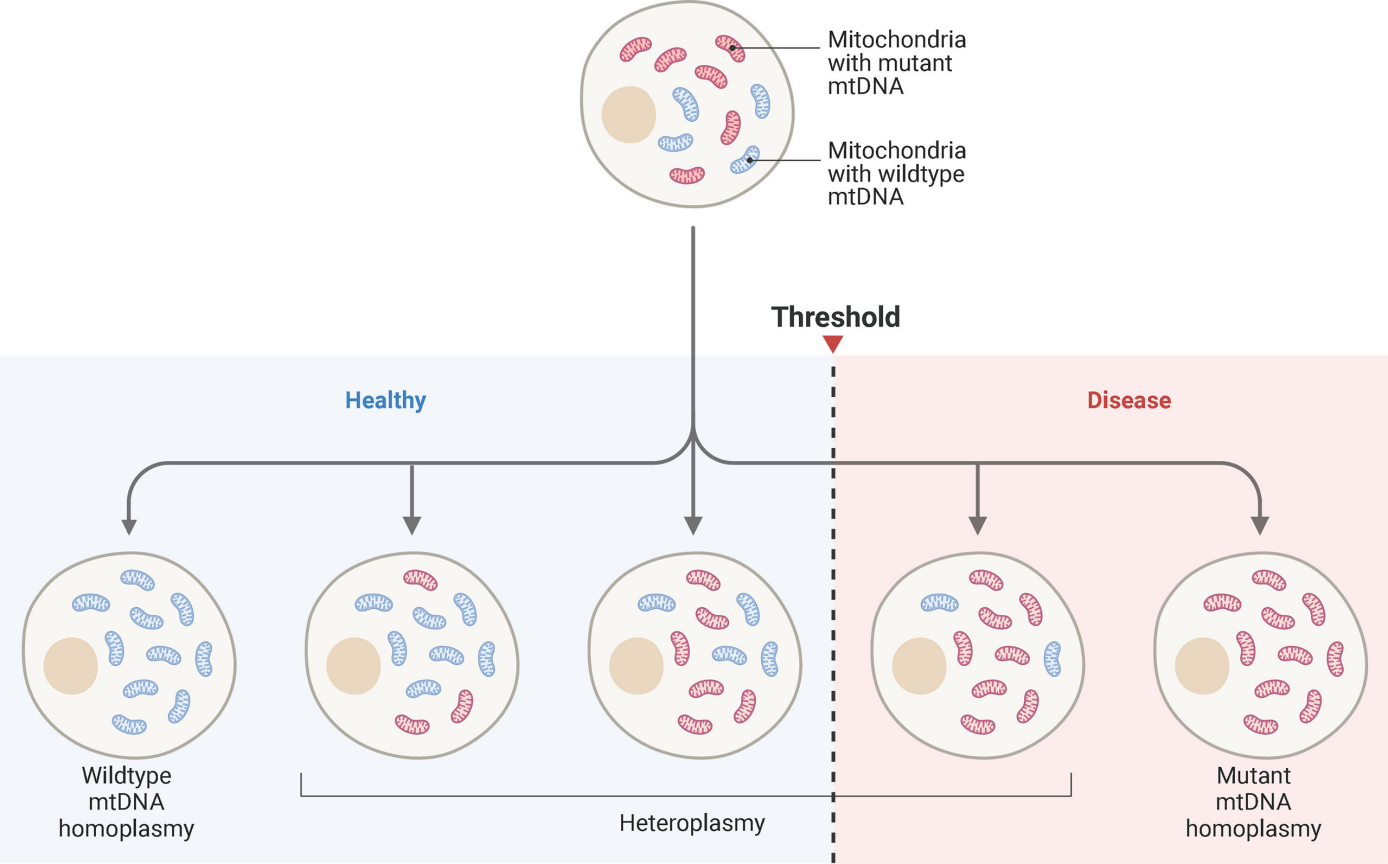
Mitochondrial Heteroplasmy and Disease ThresholdSchematic representation of mtDNA heteroplasmy and disease threshold. Blue mitochondria represent wild-type mtDNA, while red mitochondria represent mutant mtDNA. A cell may harbor all wild-type mtDNA, while others accumulate mutant mtDNA. The ratio of wild-type to mutant mtDNA can vary from cell-to-cell. When a pathogenic threshold is reached, a disease phenotype may emerge. The threshold depends on the pathogenicity of the mutation and bioenergetics the tissue. Cardiac tissue has high energy requirements, so a low mutant mtDNA may result in cellular dysfunction. *Adapted from “mtDNA Heteroplasmy”, by www.BioRender.com. Retrieved from https://app.biorender.com/biorender-templates*.

A single cell may contain hundreds of mitochondria, each with their own genome. All organelles within the cell may contain the same mtDNA sequence (homoplasmy) or a proportion of variants (heteroplasmy) (12). The level of heteroplasmy is extremely variable and can differ between cells and tissues (12). Pathogenic mtDNA mutations are often heteroplasmic, with disease phenotypes detected once mutant mtDNA percentages surpass a threshold (77) (**Figure 5**). This threshold depends on the mutation and cell type. Different tissues have different biochemical thresholds (77). For patients with Leber’s hereditary optic neuropathy (LHON), clinical presentation of disease arises when heteroplasmy levels exceed eighty percent (78). Those with the m.3243A>G mitochondrial mutation share similar threshold effects (79).

The dynamics of mtDNA mutations may explain the variability in presentation and clinical severity of KD. Thus, we believe that a mitochondrial SNP or specific haplogroup disrupts normal mitochondrial function. This mutation, however, does not exceed the mutational load for a KD diagnosis. It is only upon the second hit, described below, when clinical features of KD fully emerge.

### 4.2 Background of hit two

Disruption of mitophagy processes allows viruses and bacteria to bypass immune defenses, resulting in abnormal functioning of immune cells through exaggerated activation of NLRP3 inflammasomes and secretion of pro-inflammatory cytokines. Microbial components impair mitochondrial function; but for those with mtDNA mutations, these responses are exacerbated. Patients with mitochondrial disease commonly experience recurrent or severe infections (80), which suggests that infections play a critical role in primary mitochondrial disease and may have similar consequences for those with secondary mtDNA mutations. As such, the manipulation of host mitochondrial functions during bacterial or viral infections may explain the dysregulated immune response in KD.

A mutation in the mitochondrial genome or a functional SNP in the nuclear genome, such as *ITPKC* (81–83) which promotes NLRP3 expression, serves as the first hit, resulting in the accumulation of dysfunctional mitochondria (albeit at a level below the mutational threshold for disease). Those with the first hit are genetically susceptible to infection, which further compromises impaired mitochondria.

The second hit results in amplified activation of NLRP3 inflammasomes and proinflammatory cytokine release, which amplifies mtDNA “leakage” and mROS formation. An inflammatory cycle results and triggers the systemic vasculitis and clinical features associated with KD. We suggest both hits contribute to the development of KD.

## 5 Limitations and conclusion

### 5.1 Limitations

Mitochondria are critical in maintaining immune function. Several inflammatory diseases are associated with dysfunctional organelles, which may also amplify inflammatory responses in KD. Despite advancements in recent years, mitochondrial processes in KD remain unstudied. We propose a model of mitochondrial determinants in KD, which may elucidate current gaps in the etiology and epidemiological disparities of KD. Our hypothesis lacks specific mitochondrial data for patients with KD. Mitochondrial involvement in pediatric diseases are well documented, which are commonly inherited through maternal lineages or in an autosomal recessive pattern (84–87). Genetic factors contribute to KD susceptibility with increased incidence among parents, siblings, and extended family members (2). No clear inheritance pattern currently exists, but a population-based study in Taiwan found that children born to mothers with autoimmune diseases had a higher risk of developing KD compared to mothers who did not have an autoimmune disorder (88). Thus, a mitochondrial SNP or haplogroup passed down the maternal lineage may contribute to the first hit. Further studies are needed to determine the role of mitochondrial dysfunction in KD and potential mitochondrial DNA mutations exaggerating the immune response.

Initial treatment for KD requires timely administration of intravenous immunoglobulin (IVIG) and aspirin. Although IVIG treatment effectively suppresses inflammation in most patients with KD, about ten to twenty percent fail to respond (“IVIG nonresponders”). These IVIG-nonresponders have a higher risk of developing CA abnormalities (2). The mechanism of action of IVIG treatment still requires clarification but may coordinate with mitochondria to suppress inflammatory cytokines and antibody synthesis. IVIG neutralizes B cell survival factors (BAFF), which are required for B-cell activation and maintenance (89). Promoting secretion of proinflammatory cytokines (i.e., IL-1β), several autoimmune diseases are associated with overexpression of BAFF, including SLE (90). Mitochondria may amplify BAFF expression through increased ROS levels (91) and induce formation of the NLRP3 inflammasome (92). Thus, IVIG may target mutant mitochondria with exaggerated ROS production. However, no evidence supporting a clear relationship between IVIG and mitochondria exists. Studies exploring this potential relationship are needed to better understand the differences in treatment response, which may provide an alternative mechanism of action for IVIG.

Impaired differentiation of immune cells and subsequent metabolic reprogramming may also play a role in KD. The nature of KD suggests both T- and B-cell involvement. Administration of IVIG expands regulatory T-cell populations (2). As previously discussed, mitochondria can regulate the transcription of cells through epigenetic markers, but mitochondria also control metabolic pathways (TCA cycle, fatty acid oxidation, and OXPHOS) within immune cells (93). Although we did not discuss this role of mitochondria in-depth, Angala et al. provide a comprehensive review of mitochondrial processes in immune cells (93). Interestingly, B-cells chronically exposed to BAFF underwent metabolic reprogramming, which was essential for antibody production (94). Thus, dysfunctional mitochondria may alter metabolite availability and induce reprogramming of T- and B-cells in KD, which might be modified by IVIG treatment.

### 5.2 Mitochondria as a potential therapeutic target

Currently, patients with acute KD are treated with a single infusion of IVIG and ASA (2). However, a subpopulation does not favorably respond, and intensification of therapy is warranted. Given the potential involvement of mitochondria in KD, therapies that restore or preserve mitochondrial function, such as antioxidants, may offer an alternate or adjunct to these conventional therapies. However, studies substantiating the role of mitochondria in KD pathogenesis need to be performed before these strategies are entertained.

### 5.3 Conclusion

Further studies exploring the potential role of mitochondrial mutations in patients with KD is necessary and may provide an alternative paradigm for disease pathogenesis, clinical severity, and treatment response.

In summary, we suggest a potential role of mitochondrial dysfunction in KD, exploring the possibility of mtDNA variants as a risk factor for severity. Although much is still unknown about KD, the NLRP3 inflammasome appears to play a critical role. Oxidative damage to mtDNA may result in mutation and accumulation of dysfunctional organelles through impaired mitophagy processes and NET formation. Failure of these control mechanisms increases mtDNA “leakage”, which amplifies activation of the NLRP3 inflammasome. Initiation of the NLRP3 inflammasome leads to the release of the proinflammatory cytokines, including IL-1β and IL-18. Another feature of KD is the seasonal variation in disease onset, which is characteristic of several infectious agents. Various infections rely on manipulating mitochondrial functions to evade host immune responses, enhancing oxidative damage and mtDNA “leakage”. Further, the epidemiology of KD suggests a racial/ethnic disparity in disease prevalence. Mitochondrial haplogroups/haplotypes may explain this disparity as specific haplogroups/haplotypes are associated with various disease risks.

Even after years of research, an incomplete understanding of the pathology and etiology contributing to KD remains. Data suggests that the disease is a hyperinflammatory response to an environmental agent in genetically susceptible children. These children may have dysfunctional mitochondria prone to initiating an exaggerated inflammatory response, which is further exacerbated upon mitochondrial manipulation during infection. Exploring the dynamic interplay of impaired mitochondrial function and inflammation in KD pathophysiology is warranted.

## Author contributions

MB conceptualized the two-hit model and designed all figures, MB, MP, SS, and KS drafted and revised the manuscript. All authors contributed to the analyses presented and take responsibility for the integrity of the submitted manuscript.

## Funding

Authors are supported by NIH R01 HL 146130 awarded to MP and SS.

## Acknowledgments

Figures were created using BioRender.com.

## Conflict of interest

The authors declare that the research was conducted in the absence of any commercial or financial relationships that could be construed as a potential conflict of interest.

## Publisher’s note

All claims expressed in this article are solely those of the authors and do not necessarily represent those of their affiliated organizations, or those of the publisher, the editors and the reviewers. Any product that may be evaluated in this article, or claim that may be made by its manufacturer, is not guaranteed or endorsed by the publisher.
